# Brain Entropy Mapping Using fMRI

**DOI:** 10.1371/journal.pone.0089948

**Published:** 2014-03-21

**Authors:** Ze Wang, Yin Li, Anna Rose Childress, John A. Detre

**Affiliations:** 1 Department of Psychiatry, Perelman School of Medicine, University of Pennsylvania, Philadelphia, Pennsylvania, United States of America; 2 Department of Radiology, Perelman School of Medicine, University of Pennsylvania, Philadelphia, Pennsylvania, United States of America; 3 Department of Neurology, Perelman School of Medicine, University of Pennsylvania, Philadelphia, Pennsylvania, United States of America; Wake Forest School of Medicine, United States of America

## Abstract

Entropy is an important trait for life as well as the human brain. Characterizing brain entropy (BEN) may provide an informative tool to assess brain states and brain functions. Yet little is known about the distribution and regional organization of BEN in normal brain. The purpose of this study was to examine the whole brain entropy patterns using a large cohort of normal subjects. A series of experiments were first performed to validate an approximate entropy measure regarding its sensitivity, specificity, and reliability using synthetic data and fMRI data. Resting state fMRI data from a large cohort of normal subjects (n = 1049) from multi-sites were then used to derive a 3-dimensional BEN map, showing a sharp low-high entropy contrast between the neocortex and the rest of brain. The spatial heterogeneity of resting BEN was further studied using a data-driven clustering method, and the entire brain was found to be organized into 7 hierarchical regional BEN networks that are consistent with known structural and functional brain parcellations. These findings suggest BEN mapping as a physiologically and functionally meaningful measure for studying brain functions.

## Introduction

Entropy indicates system irregularity [1], which remains relatively low in living systems but increases over time in any closed system like our universe as dictated by the second law of thermodynamics [1], [2]. As nearly the most complex living organism known to us, the human brain has a prominent need for sustaining its entropy to function normally [3]–[5]. Measuring brain entropy (BEN) might then provide a physical means for characterizing brain status as well as its alterations in disease. BEN has long been measured using electrophysiological data [6]–[10] with low spatial resolution. Functional MRI (fMRI) reflects regional changes in cerebral blood flow and metabolism [11] and provides time-resolved volumetric imaging of brain function at relatively high spatial resolution, making it an attractive approach for mapping BEN. In the context of fMRI, entropy was first assessed as a novelty index [12], then as a tool for activation detection [13], and recently as a potential marker for brain diseases using the resting-state fMRI (rsfMRI) data from prior-selected regions-of-interest (ROIs) [14], [15]. Entropy of the entire brain has also been assessed using fMRI [16]–[18], but little is known about BEN's spatially distribution and its regional organizations. This study is an extension of our previously presented preliminary BEN work [19]–[21]. The purposes of this paper were to generate a whole brain entropy map using a large cohort of subjects (n = 1049) and to investigate the spatial distribution and local organization of BEN.

As directly calculating entropy from fMRI data is challenging and inaccurate due to the difficulty of accurately estimating the probability distribution function from the moderate number of time points contained in a typical fMRI time-series, we used sample entropy (SampEn) [22], [23] to measure entropy of fMRI timeseries, which is an approximate entropy measure that is stable for different data lengths and across different sessions [22], [23].

In tandem with BEN mapping, we sought to examine the regional BEN structure and to develop a brain atlas based entirely on the entropic correlations of brain regions, rather than using *a priori* structural or functional information. Our hypothesis was that BEN would discriminate between neuronal and non-neuronal (phantom-based) dynamics. Furthermore, we hypothesized that it would show a structured regional specificity based on empirical observations about slow coherent fluctuations of activity in fMRI – and theoretical work suggesting a hierarchical organization of temporal dynamics in the cortex [24]. To evaluate the SampEn-based BEN mapping and to test the above hypothesis, we first performed a series of control experiment to verify both the sensitivity and specificity of SampEn, and then compared fMRI derived entropy of a static phantom and the normal brain as well as the test-retest stability of BEN mapping in normal brain, and then conducted BEN mapping in normal brain using the large sample size from the 1000 functional connectome project (FCP) database, followed by BEN clustering and regional organization investigation.

## Materials and Methods

### Human subjects

16 young healthy subjects (age = 25.4±4.5, range = 20.3–35, male/female: 8/8) were recruited from University of Pennsylvania with approval from local ethics committee and signed consent forms from the participants. rsfMRI and fMRI with a sensorimotor task were acquired from the 16 subjects twice with 2 months apart. Detailed recruiting criteria and imaging parameters can be found in [25], [26]. The same fMRI sequence was also used to acquire rsfMRI data from a water phantom.

Resting fMRI were then downloaded from the 1000 functional connectomes project (FCP) [27]. All FCP data were acquired and released with approval from each contributor's ethics committee. Data from 22 centers (n = 1175) were analyzed, and those with small brain coverage or without demographic information (age and gender) were excluded, resulting in a total of 1049 subjects (age = 26.94±11.34 (mean ± standard deviation (STD)) years, age range: 7.88∼85 years, 466 males, 583 females) with rsfMRI data and high resolution anatomical image. 50 subjects had repeat data. The acquisition parameters were: duration: 4.15∼9.8 min; voxel size: 2∼4 mm within plane; slice thickness, 3∼5.5 mm.

### Sensorimotor task

The task consisted of 5 resting and activation blocks, each block lasting for 48 seconds and was presented using Presentation (Neurobehavioral Systems, Albany, CA, USA). During the task block, visual stimuli with an 8 Hz reversing black and white checkerboard were presented intermittently in the full visual field for 48 seconds, and the subject was asked to perform a self-paced left-hand only fingertapping task when they saw the visual stimuli.

### Data acquisition

For the 16 subjects, structural imaging and rsfMRI acquisitions were described in [25], [26]. Sensorimotor fMRI was acquired using the same sequence as that of rsfMRI. The same structural MRI, rsfMRI, task fMRI were repeated in 2 months. The same fMRI sequence was also used to acquire rsfMRI data from a water phantom.

### Image processing

Image preprocessing was performed using AFNI (afni.nimh.nih.gov/afni/) and FSL(www.fmrib.ox.ac.uk/fsl/) with standard processing steps [27] including motion correction, temporal filtering, and spatial smoothing. Nuisance cleaning were performed as described in [27], [28], but without global signal cleaning [29]. We used the Nonlinear Image Registration Tool [30] to register each subject's structural image into the MNI standard space. Statistical analysis was performed using SPM (http://www.fil.ion.ucl.ac.uk/spm/).

### Entropy calculation

Directly calculating entropy from fMRI data is challenging and inaccurate because of the difficulty of accurately estimating the probability distribution function from the moderate number of time points contained in a typical fMRI time-series. To overcome this challenge, we used sample entropy (SampEn) [22], [23], which is an extension of Approximate Entropy (ApEn) [31], both are approximations to Kolmogorov complexity/entropy [32]. SampEn is shown to be stable for different data lengths and across different sessions [22], [23]. Since SampEn needs to be calculated for hundreds of thousands of voxels for the >1000 subjects, we used an optimized SampEn algorithm implemented in C++ computer language to reduce the total computation time.

Denote the rsfMRI data of one voxel by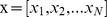, where N is the number of time points. SampEn starts with forming a series of vectors, the so called embedded vectors, each with m consecutive points extracted from x: 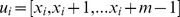, where 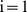 to N-m+1, and m is the pre-defined dimension. Using a pre-specified distance threshold r, 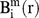 counts the number of
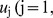
to N-m, and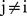) whose distances (Chebyshev distance is generally used though any other distance can be used as well) to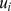 are less than r, so does
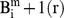
for the dimension of m+1. By averaging across all possible vectors, we have
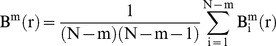
(1)

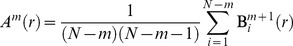
(2)And SampEn is calculated as:
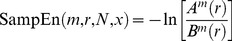
(3)In the original algorithm, the distance of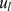 and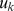 and the subsequent comparison with r are conducted twice: one for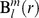 and the other for 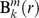. This redundancy was removed by updating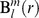 and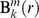 simultaneously and flagging these operations for this pair to be excluded in later iteration. For fMRI data with N time points, this routine will save (N-m-1)*(N-m-2)/2 times distance calculations and (N-m-1)*(N-m-2)/2 times distance comparisons. Meanwhile, the first m elements of the embedded vectors of dimensions m and m+1 are the same, meaning that distance calculations for m can be shared with m+1, while the latter will only needs to be updated with the last element of the vectors. This SampEn calculation algorithm was verified with Matlab code implementing the original algorithm to make sure that it yielded the same results as the original implementation.

### SampEn evaluations

As described above, SampEn depends on an embedded dimension m and a tolerance threshold r and different m and r may yield different SampEn values. Small m and large r lead to more vector matches (higher 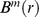 and 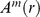 in Eqs. 1 and 2. Note that smaller m tends to yield smaller distance) and subsequently improve the accuracy and confidence level of SampEn. However, the definition of SampEn requires that m approaches ∞ and as r approaches 0 [31]. To verify SampEn with different m and r for differentiating signals with different known entropy and also to find an empirically optimal value for both parameters, SampEn calculations were performed with different m and r using synthetic data with known entropy.

### Evaluation 1

SampEns of Gaussian noise, chirp signal, and sinusoidal signal (Fig. S1A) were calculated with different r and m. Based on the literature [23], [33]–[35], in the simulations, m was changed from 3 to 4; r was changed from 0.2 to 1.2 with a step of 0.2 and then changed to 1.5 of data STD. The length of these synthetic data was set to be 150 to 400.

### Evaluation 2

As described in [36], [37], synthetic fMRI data with a periodic on-off paradigm were generated with 6 different contrast-to-noise-ratios (CNRs): 0.08, 0.1, 0.2, 0.5, 0.8, and 1. Synthetic data were generated at each noise level by adding new Gaussian noise for 100 times. Both noise and activation were convolved with the canonical hemodynamic reference function (HRF) [38] to mimic the real fMRI noise environment. Fig. S1C shows the HRF convolved design function (red dashed line) and the noise contaminated signal with a CNR of 0.2 (blue solid line). SampEn was calculated for both noise and synthetic activation data using m from 3 to 4, and r from 0.2 to 1 STD.

### BEN mapping evaluations

**Figure 1:**
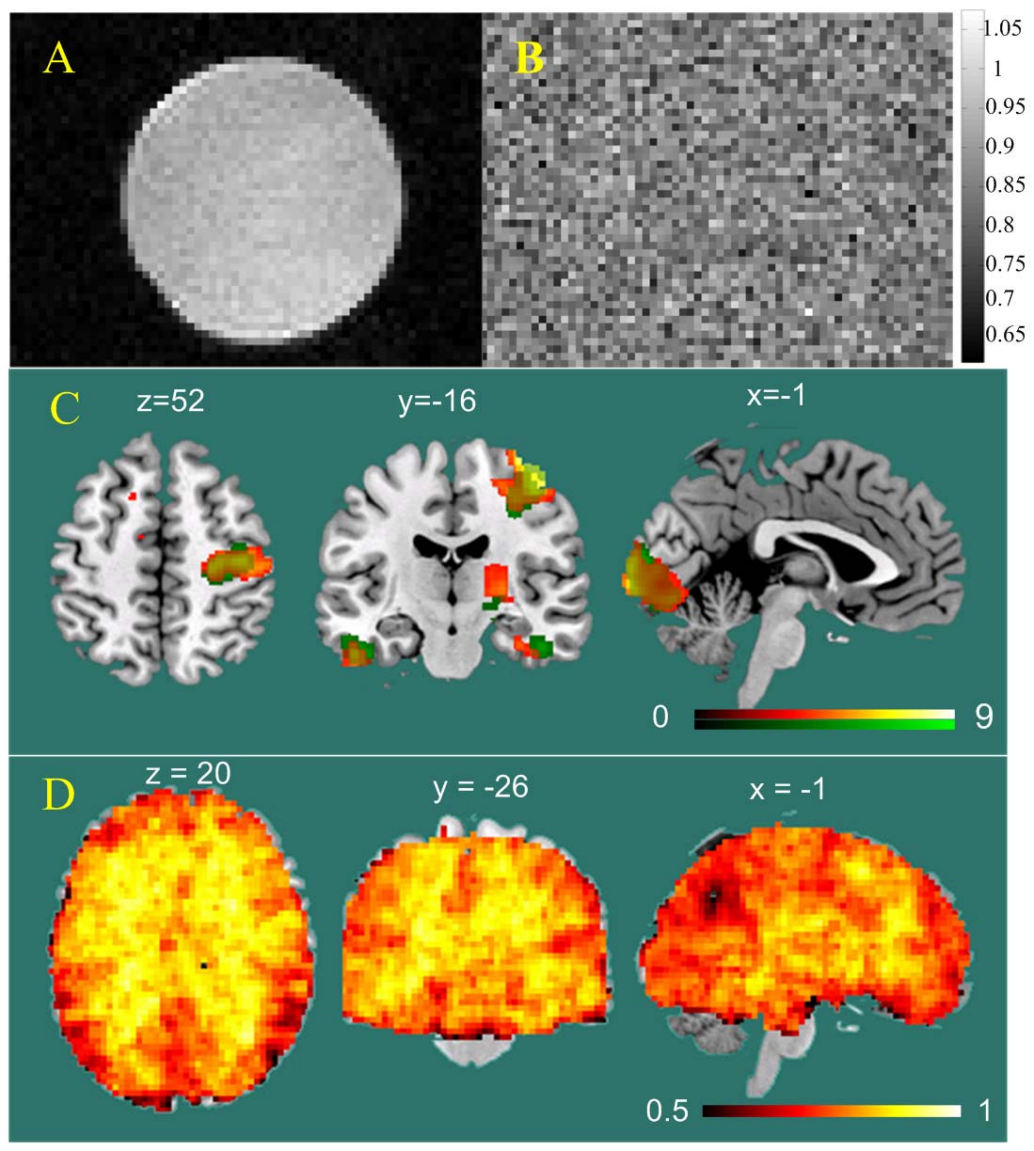
Evaluations of entropy mapping using fMRI. A) image of a cylinder water phantom; B) entropy map of the phantom. The color bar indicates the display color window for the entropy map; C) visual and sensorimotor functional activation induced brain entropy decrease as compared to the resting state. Red and green represent the test (session 1) and retest (session 2) experiment, respectively. The color bars indicate the color window used to display the statistical comparison results (t maps of the paired student t-testing). The statistical threshold for identifying the entropy decreasing clusters is p<0.001 and cluster size>30 (uncorrected for multiple comparisons); D) the correlation coefficient (CC) map of 50 subjects' resting BEN maps. The color bar was used to map the CC value from 0.5 to 1.

After the above SampEn evaluations, we performed several control experiments to validate BEN mapping using fMRI. The first control experiment was to check the entropy level in a nonliving water phantom (Fig. 1A shows one image slice of the phantom). Entropy of each voxel within and outside of the phantom was calculated using the SampEn algorithm described above and the non-processed raw fMRI data. Since fMRI signal of the static phantom consists of random thermal fluctuations and a random low frequency drift [39] with high irregularity, entropy of the phantom should be no different from that of the background noise. The second control experiment was performed to verify the test-retest reproducibility of BEN mapping results in normal brain. To do that, SampEn was calculated at each voxel of the preprocessed rsfMRI data from 50 subjects identified from the FCP database who had repeated rsfMRI scans. The collection of all voxels' SampEn formed a BEN map, which was warped to the MNI standard space using FNIRT. The intra-class correlation coefficient (ICC) [40] of BEN at the test and retest sessions was calculated at each voxel using Matlab. Correlation coefficient (CC) of the whole brain mean BEN of both sessions was also calculated.

The third experiment was to assess whether BEN mapping can capture the brain activity irregularity changes during performing periodic sensorimotor task. Entropy has been assessed as a way to detect brain activation in [13], but was derived from a small window around the assumed brain activation epoch peaks. In this paper, entropy of the entire fMRI time series was characterized. In this experiment, we calculated BEN maps from the 16 subjects' rsfMRI data and sensorimotor fMRI data which were separately acquired in the same scan session and were repeated in another session in 2 months. Since brain activity in response to the periodic task performing would introduce regional orderliness to the fMRI data, regional BEN should decrease accordingly. SampEn was calculated at each voxel of the preprocessed rsfMRI and task fMRI images. The resulting BEN maps were registered and resampled to a resolution of 3×3×3 mm^3^ into the MNI space using FNIRT. A paired t-test between BEN maps of rsfMRI and task fMRI was conducted for each scan separately to assess the regional BEN alterations due to the periodic task performing.

### BEN mapping using the FCP data

BEN maps were calculated using the routines described above. Each BEN map was divided by its mean and subtracted by 1 to get an rBEN map. Using FNIRT, both BEN and rBEN maps were projected into the MNI space with a resolution of 3×3×3 mm^3^. We then calculated the mean BEN maps of all 1049 subjects and conducted a one-sample t-test on the rBEN maps. Age and gender effects were included in a multiple regression model to assess their effects. This regression was conducted on the whole brain mean BEN and each voxel's BEN separately.

### Clustering

To further delineate the entropic patterning of the brain to test the hypothesis that BEN is self-organized into regional communities, we used a data-driven clustering method to automatically parcellate whole brain rBEN values into a series of clusters. We used rBEN maps to remove across-subject global BEN variability. To reduce the computational burden for the whole brain based BEN clustering, rBEN maps were resampled with a resolution of 4.5×4.5×4.5 mm^3^ in MNI space. We used a fast and robust clustering algorithm, spectral clustering [41], to search for regional BEN communities (clusters). We chose spectral clustering which has been shown to have good performance in various applications [41] including fMRI-based brain segmentation [42]. With 1049 subjects' BEN maps, we had an nxS BEN data matrix, where n = 15893 is the number of intracranial voxels, *S* = 1049 is the number of subjects. Spectral clustering starts with building an nxn similarity (CC in this study) matrix. With the similarity matrix, a Laplacian matrix was built. Using eigen decomposition, the k smallest eigenvectors of the Laplacian matrix were extracted and grouped into an Sxk matrix, which was subsequently normalized along each row. Treating each row as a new data point, standard k-mean clustering was used to cluster the S points into k clusters. k was the pre-specified cluster number. We used the Matlab code written by Ingo Buerk (http://www.mathworks.com/matlabcentral/fileexchange/34412) to do spectral clustering using the algorithm proposed by [43].

### Determining the optimal cluster number

Since clustering produces the results based on the pre-specified cluster number, we performed a series of experiments to determine the optimal cluster number. Clustering was performed for the entire group and a randomly assigned 525 group and a 524 group with k varying from 3 to 40. For each k, we calculated Silhouette coefficient (SC) [44] and a reproducibility index (RI). RI is the number of non-reproducible clusters across the 3 groups. For each k, we got 3 sets of clusters. For each of the k clusters from the 1049 group, we calculated its overlap ratio with each of the k clusters of the two subgroups (the 525 and 524 group). The overlap ratio of two clusters was the ratio of the number of overlapped voxels divided by the total number of voxels in both clusters. The cluster in each of the two subgroups showing the highest overlap ratio to the target cluster in the 1049 group was identified as its partner. We set the partner to be null if no cluster in that subgroup showed greater than 10 percent of overlap ratio with the target cluster of the 1049 group. We then calculated the number of mis-matched clusters by counting the number of null partners of all k clusters of the 1049 group. We repeated the same partner-matching procedure for all clustering experiments with k varying from 2 to 40. We then used the number of mis-matched clusters as the RI for the k-clusters-based clustering. Fewer mis-matchings means higher RI. The optimal k was identified at the peak (local maximum) of SC curve and the peak (local minimum) of the RI curve.

### Rank and hierarchy of regional BEN

This experiment was to further evaluate the possible hierarchical structure of the BEN communities identified in the above experiment. We extracted the mean rBEN for the above identified clusters from all subjects. One-way ANOVA function and a series of paired t-tests were performed to test whether there are significant rBEN differences among those clusters. The entropy similarity matrix was also created using the mean cluster rBEN series (across subjects), a dendrogram was generated using the hierarchical clustering method provided in Matlab (Mathworks, Natick, MA, USA) to summarize the entropy relations of the regional entropy communities identified above.

## Results

### SampEn with different m and r for differentiating signal from noise

Simulations on SampEn calculations showed that data length has only a minor effect on SampEn (**Fig. S1B**), which ensured including all the rsfMRI data even with different time points for BEN mapping. For fMRI, SampEn robustly detected the synthetic brain activity from the noise contaminated data even when the contrast-to-noise-ratio was as low as 0.2 (see **Fig. S1D** and **Table S1**), though its sensitivity decreases when r was greater than 1. Since SampEn with m = 3 or 4 showed similar performance for differentiating signal from noise (**Fig. S1D**) and small m may improve SampEn-based entropy calculation accuracy, m = 3 was chosen as the empirical value for rsfMRI-based BEN mapping. When r was greater than 0.6 STD, different m's yielded similar SampEn values and the SampEn difference between the Sinusoidal and chirp signal stayed at the same level. Therefore, r = 0.6 STD was chosen to be the optimal value for the following BEN mapping.

### Brain entropy mapping using fMRI

In the first fMRI-based entropy mapping experiment, the water phantom (Fig. 1A) showed the same entropy as that of the background except random spatial fluctuations (Fig. 1B). In the second evaluation experiment, sensorimotor task performing introduced significant (p<0.001 and cluster size>30, uncorrected for multiple comparisons) regional SampEn decrease in visual and sensorimotor brain areas as compared to the resting state (Fig. 1C). The SampEn decrease due to task performing in scan day 1 (the hot spots) was replicated in a second experiment performed 2 months later (the green spots). Fig. S2 shows the brain activations identified using standard GLM. The GLM results confirmed the SampEn identified functional regions in both sessions. Fig. 1D shows the test-retest intra-class correlation coefficient (ICC) map of the 50 FCP subjects' BEN maps. Nearly all intracranial voxels showed an ICC>0.5. ICC for the whole brain mean BEN was 0.967. Several voxels in the posterior cingulate cortex showed an ICC<0.5.

### Normal BEN distribution revealed using 1049 normal subjects

**Figure 2:**
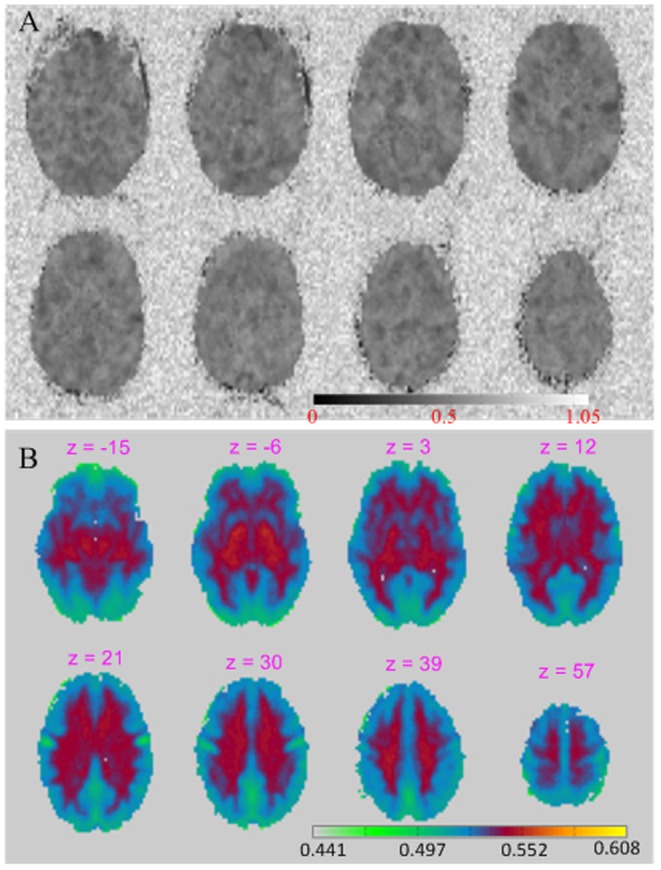
The BEN maps with and without background. A) BEN map of a representative subject. B) average BEN map of 1049 subjects. The background voxels were removed for a better visualization.The gray scale and color scale indicate BEN values for Figure 2a and 2b, respectively.

Fig. 2A shows the BEN map (calculate using SampEn with m = 3 and r = 0.6) from a representative subject from the FCP database. Similar to the result shown in Fig. 1B, entropy in the background is high and randomly distributed. However, BEN in the intracranial voxels appears to be much lower than the background.

**Figure 3:**
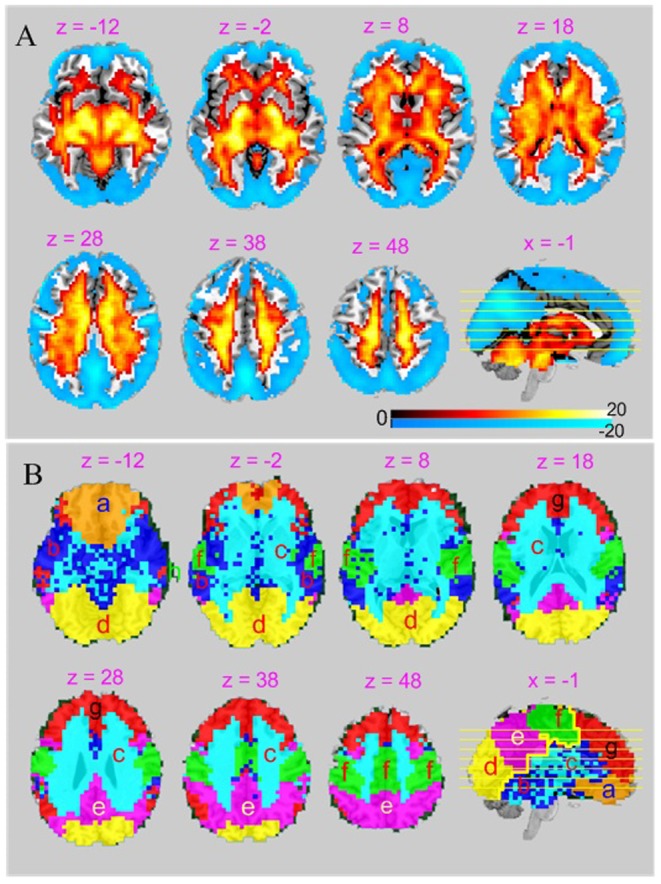
Spatial distribution and regional organization of BEN. A) The statistically defined higher-than-average BEN network (hot color) and lower-than-average BEN network (cool color). The significance level used for thresholding the distribution map (the statistical parametric map of the student t-test) is p<0.01 (corrected for multiple comparison); B) The 8 BEN clusters identified by clustering: a) OFC, b) TPLS, c) WM, d) VIS, e) DMN, f) MC, g) PFC, h) peripheral artifact. The text above each axial slice in B, C, and D indicates the slice location (z and x mean the x and z coordinate in mm, respectively) in the MNI space. The left side of image corresponds to the left side of brain.

Fig. 2B shows the mean BEN map of the 1049 subjects with background removed for better visualization of the detailed BEN structure (very similar patterns were found in the mean BEN maps calculated with different r values as shown in Fig. S3). The mean BEN map shows a clear BEN contrast between neocortex, white matter (WM), and subcortical gray matter structures. Neocortex showed lower BEN than the rest of brain, with the 5 lowest BEN regions located in the precuneus (PRE), bilateral motor cortex (MC), orbito-frontal cortex (OFC) and visual cortex (VIS) (Fig. 2B, 3A). Low entropy in PRE, OFC, and VIS is consistent with prior results [14]. Whole brain BEN did not show age effects, either regionally or globally. By comparing rBEN to 1, we statistically (p<0.01, corrected for multiple comparison) defined the high and low BEN distribution pattern shown in Fig. 2B as a higher-than-average BEN network (HBEN) and a lower-than-average BEN network (LBEN) in Fig. 3A.

### The hierarchical self-organization of BEN

On the basis of the peak of the SC (Silhouette coefficient) [44] and the peak of a clustering reproducibility index (RI) (Fig. S4), we identified 8 BEN clusters (Fig. 3B). One cluster (*h* in Fig. 3B) consists of voxels within the peripheral brain boundaries, likely to represent artifact due to imperfections in brain registration. The other 7 clusters correspond to anatomically or functionally meaningful regions. LBEN identified in Fig 3A was reorganized into 5 BEN clusters (a, g, f, e, and d in Fig. 3B) located in OFC, prefrontal cortex (PFC), MC, the posterior part of the “default mode network” (DMN) [45], and VIS, respectively; while HBEN was separated into clusters b, and c, where c consists of WM, and b covers cerebellum, brain stem, limbic area, inferior temporal cortex (ITC), and part of medial temporal cortex (MTC) (cluster b will be called temporal lobe-subcortex (TPLS) cluster in the following). All clusters were bilateral, albeit with some asymmetry. By randomly splitting the whole group into two subgroups with n = 525 and n = 524 respectively, we verified that the 8 cluster parcellation results for the n = 525 subgroup (Fig. S5A) and the n = 524 subgroup (Fig. S5B) are highly reproducible.

**Figure 4:**
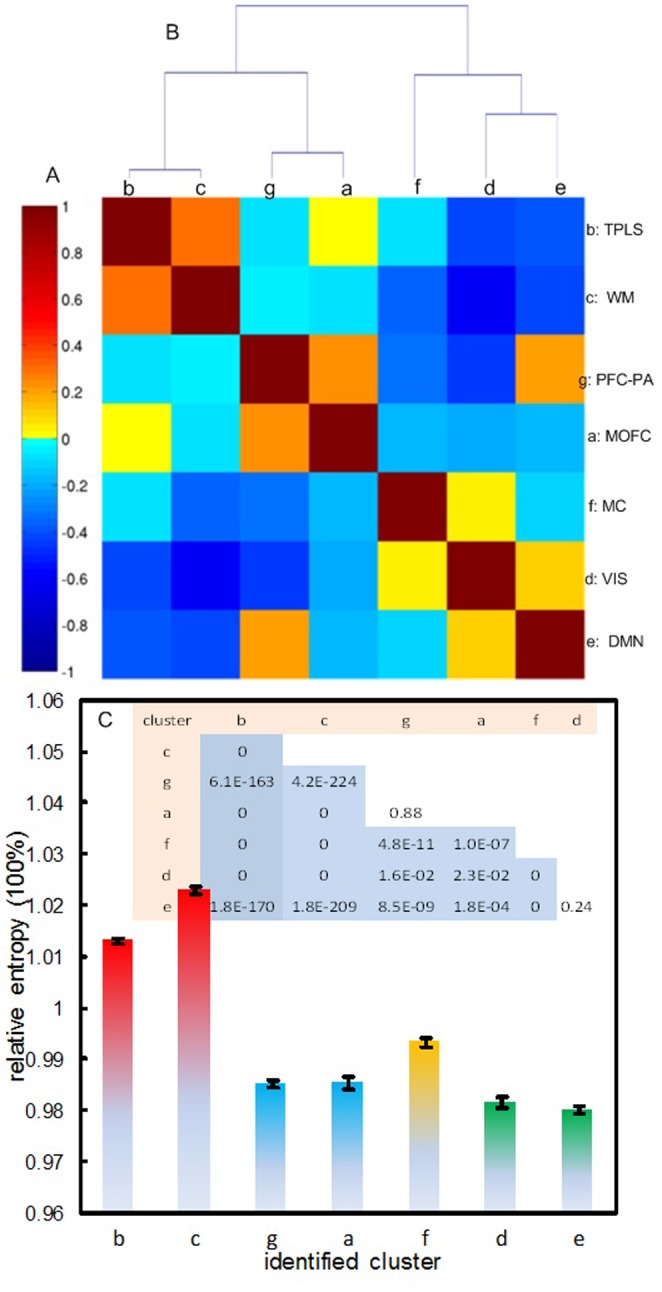
The hierachy and ranking of the BEN-derived brain subdivisions. A) the across-subject BEN similarity matrix, B) the hierarchical structure of the 7 BEN clusters. C) Mean and standard errors of the mean rBEN in the 7 clusters. The color map in A represents the correlations of BEN within and between BEN clusters. The inset in C lists the probability of the rBEN comparisons between a pair of BEN clusters.

Fig. 4A shows the entropy similarity matrix of the 7 structurally and functionally meaningful clusters identified above. Fig. 4B is the dendrogram generated with the hierarchical clustering. The most distinct BEN partitions at the highest level of the hierarchy corresponds to a division between an anterior BEN system (PFC, OFC, WM, and TPLS (g, a, c, and b in Figs. 3B, 4) and a posterior BEN system (MC, DMN, and VIS (f, e, and d in Figs. 3B, 4) as marked by the thick yellow line in the sagittal view of Fig. 3B; below the anterior BEN system are the frontal BEN network (PFC and OFC) and HBEN, while below the posterior BEN system are the visual-DMN system (VIS and DMN) and the sensory motor system (MC).

Using the one-way ANOVA, we found that the 7 clusters identified above showed significantly (p = 0) different rBENs (Fig. 4C). Among them, the WM cluster showed the highest BEN, followed by TPLS (cluster b in Fig. 3B), MC, PFC, OFC, VIS, and DMN (Fig. 4C). PFC and OFC showed the same level of rBEN, and no rBEN difference was found between VIS and DMN. Significant rBEN differences were demonstrated between each pair of the remaining 5 cluster groups: 1) WM, 2) TPLS, 3) MC, 4) PFC and OFC, 5) VIS and DMN (see the inset for the paired-t test probability values Fig. 4C).

## Discussion

We mapped whole brain entropy using resting state fMRI from 1049 normal subjects. Entropy was measured with SampEn. Our data demonstrated that SampEn is a robust and sensitive entropy measure for differentiating generic and fMRI signals with different regularities and that SampEn-based fMRI-derived BEN is reliable brain activity measure, which differs from that of a non-living object and background noise, and is sensitive to task-induced regional brain activity alterations. BEN was stable in nearly the whole brain except several voxels in the middle sagittal plane. While their ICC were still >0.4, the relatively lower test-retest stability may reflect that resting state activity in those voxels which are in the precuneus has larger across-session variability.

Low entropy has long been postulated and observed in living organisms [2] including human brain [3]–[5]. Our results clearly demonstrated the big entropy contrast between the living brain tissue and the background and the nonliving water phantom, which retrospectively proves physical validity for the fMRI-based BEN mapping method, and is consistent with Lovelock's suggestion of using entropy for detecting life in Mars which was further developed into the Gaia theory [46], [47]. The whole brain entropy map showed a clear spatial distribution and a sharp contrast between neocortex and the rest of brain. The relatively lower BEN in neocortex may reflect the “higher” mental functions subserved by cortex [48]. Within the neocortex, low entropy in PRE (precuneus), OFC (orbito-frontal cortex), and VIS (visual cortex) is consistent with prior results [14] where BEN was measured in anatomical ROIs using wavelet entropy [49] and no statistical inference was provided for BEN distributions across the brain. The white matter versus grey matter BEN contrast was consistent with our previous findings using a negentropy-based BEN mapping method [19], [20] and those reported in [18] which was based on ApEn [31].

The heterogeneity of neocortex BEN and its organization into regional communities are indirectly supported in prior rsfMRI studies [45], [50]–[52]_ENREF_35. Specifically, PRE and OFC are within the so-called DMN [45], which is known to fluctuate in a coherent way [27]. MC has long been demonstrated to have strong correlations between its bilateral segments by Biswal et al in their seminal functional connectivity paper [51]_ENREF_41. VIS has also been repeatedly identified as a separate resting state network [50]. BEN clusters were bilateral, suggesting no side dominance of resting BEN across subjects. BEN clustering based brain parcellation is purely driven by the data. Although we controlled the stability of BEN clustering by repeating the process to randomly selected sub-data sets and presented the results that had both a local maximal SI coefficient and a local maximum of reproducibility, it is still possible that new data might give different number of optimal clusters. However, finding a different number of clusters should not change the general patterns of the subdivisions except cluster splitting or merging as shown in the additional clustering results derived at two sub-optimal SI and RI peaks with k = 6 and 14, respectively.

The hierarchical BEN clustering findings were partly supported by recently published cortical surface area-based genetic subdivision study [53] where a similar anterior/posterior brain subdivision was reported. The hierarchical structure of the identified regional BEN clusters was further evidenced by the progression pattern shown in the 6, 8, and 14 clusters-based parcellations results (Fig. 3 and Fig. S6) that the successive clusters (when k increased) tend to be sub-partitions of previous clustering results (of smaller k).

An approximate negentropy [54] was used in our preliminary studies as reported in [19], [20]. Though the entropy contrast between grey matter and white matter shown there was similar to what we found in this paper, the negentropy-based BEN mapping using the same FCP data showed relatively higher entropy rather than lower entropy in the motor cortex than the rest of brain. We later found that the artificial “higher” entropy in the resting motor cortex was caused by the sensitivity of the negentropy measure to input data normalization, which is not an issue for SampEn because SampEn uses a data-dependent threshold (r) for counting the vector matchings and therefore is data scale-free. Choosing r and m has been extensively performed in previous literature. We repeated the evaluation process in order to provide guidance for choosing them for BEN mapping. Though we used r = 0.6 and m = 3 in this paper, we did run a series of analyses using different values and found very similar results for those values assessed in the evaluation experiments.

It's worth to note that SampEn and ApEn are not the same as Shannon entropy. SampEn and ApEn are rather often used to indicate system complexity because both of them were defined as approximates to the Kolmogorov complexity. In our BEN mapping experiments using the sensorimotor fMRI data, we showed that SampEn reduced when brain activity turned to be more coherent due to the periodic task engagement. Since the periodic task activation would increase entropy if measured by Shannon entropy but decrease complexity, a reduction of SampEn suggests that SampEn can be potentially used as a complexity measure for brain activity. However, SampEn has not been fully shown to a complexity measure similar to the Tononi complexity [55]. In [56], a multi-scale SampEn was proposed, which showed lower SampEn in random noise than in 1/f noise when scale increased. Since 1/f noise is known to have higher complexity than random noise, the multi-scale SampEn could potentially provide a way to approximately measure signal complexity. A difficulty of using higher scale in fMRI is that increasing scale will exponentially reduce the available data length, resulting in a non-stable SampEn measurement. Since increasing scale is similar to a low-pass filtering, an alternative approach to perform multi-scale SampEn is to combine low-pass filtering and SampEn. However, that is out of the scope of this study and should be assessed in future work.

To summarize, BEN provides a physiologically and functionally meaningful brain activity measure. Different from the relative metrics used in prior rsfMRI studies, entropy is a quantitative measure, making it a potentially useful index for studying different brain states. The hierarchy of BEN suggests that brain activities can be ranked by BEN, which may provide opportunities to detect brain disorders based on abnormalities in BEN activity such as a pilot investigation of BEN in drug addiction [57].

## Supporting Information

Figure S1 **SampEn evaluation results.** A) waveforms of a Gaussian noise, a chirp signal, and a sinusoidal signal used for evaluating SampEn; B) SampEn calculation results using different embedded dimensions (m) and different tolerance levels (r) with different data length; C) the hemodynamic response function (HRF)-convolved artificial brain activation (the red line) and noise (the blue line); D) SampEn of noise (blue lines) and noise contaminated brain activation (red lines). The errorbars indicate standard error of SampEn for each calculation.
(DOCX)

Figure S2 **Brain activations identified using standard general linear model.** The task activation was defined as the significant brain activity magnitude between the task condition and control condition. The significance level used here is the same as that in Fig. 1C. Red and green indicate results of the first and second day sensorimotor task performing experiment. Colorbar means the t-value range of the statistical parametric maps shown here.
(DOCX)

Figure S3 **Mean resting BEN maps of 1049 subjects calculated using SampEn with m = 3, r = 0.4, 0.6, 0.8, 1 from the bottom row to the top row, respectively.** For the purpose of display, every BEN map has been normalized to be from 0 to 1 (divided by its maximum intensity). The colormap shows the display window used for generating the maps and its range is from 80% to 101% of the maximum.
(DOCX)

Figure S4 **Silhouette coefficient (red line) and the number of non-reproducible clusters (blue line) of BEN clustering using different pre-specified cluster numbers.**
(DOCX)

Figure S5 **Two different data subsets-based BEN clustering results.** A) Eight brain subdivisions identified from the 525 randomly selected subjects' rBEN maps, B) eight brain subdivisions the rest 524 subjects' rBEN maps using the data-driven spectral clustering method. These clusters were identified at the optimal peak of the curves shown in Fig. S4 using the same spectral clustering procedure as described in the main article.
(DOCX)

Figure S6 A) six brain subdivisions and B) fourteen brain subdivisions derived from 1049 subjects' rBEN maps. These clusters were identified using the same spectral clustering procedure as described in the main article. The prior specified cluster number k of 6 and 14were located in the two suboptimal peak locations adjacent to the optimal one k = 8 as shown in Fig. S4.
(DOCX)

Table S1 **Signal detection results using sample entropy and reference guided data fitting.** Synthetic data were generated by adding noise to a boxcar function with 8 different level of contrast-to-noise-ratio (CNR). SampEn was calculated using an embedded dimension of 4, and 5 different tolerance levels. cc means Pearson's correlation coefficient.
(DOCX)
