# Supplementary material for: Brain Entropy Mapping Using fMRI
Source: PLoS One. 2014 Mar 21;9(3):e89948. doi: 10.1371/journal.pone.0089948 (PMC3962327; doi:10.1371/journal.pone.0089948)
Supplement: Figure S1 — SampEn evaluation results. A) waveforms of a Gaussian noise, a chirp signal, and a sinusoidal signal used for evaluating SampEn; B) SampEn calculation results using different embedded dimensions (m) and different tolerance levels (r) with different data length; C) the hemodynamic response function (HRF)-convolved artificial brain activation (the red line) and noise (the blue line); D) SampEn of noise (blue lines) and noise contaminated brain activation (red lines). The errorbars indicate standard error of SampEn for each calculation. (DOCX) [file pone.0089948.s001.docx]

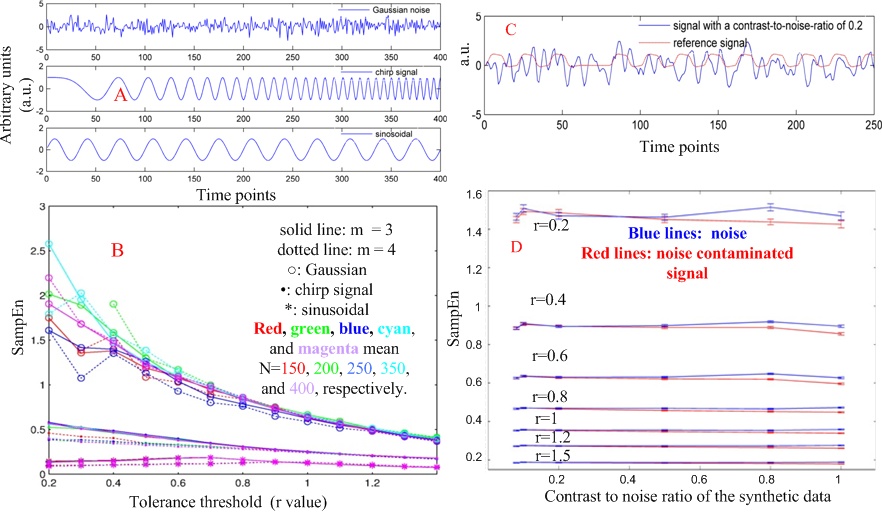


Fig S1. SampEn evaluation results. A) waveforms of a Gaussian noise, a chirp signal, and a sinusoidal signal used for evaluating SampEn; B) SampEn calculation results using different embedded dimensions (m) and different tolerance levels (r) with different data length; C) the hemodynamic response function (HRF)-convolved artificial brain activation (the red line) and noise (the blue line); D) SampEn of noise (blue lines) and noise contaminated brain activation (red lines). The errorbars indicate standard error of SampEn for each calculation.
