# Supplementary material for: Brain Entropy Mapping Using fMRI
Source: PLoS One. 2014 Mar 21;9(3):e89948. doi: 10.1371/journal.pone.0089948 (PMC3962327; doi:10.1371/journal.pone.0089948)
Supplement: Figure S2 — Brain activations identified using standard general linear model. The task activation was defined as the significant brain activity magnitude between the task condition and control condition. The significance level used here is the same as that in Fig. 1C. Red and green indicate results of the first and second day sensorimotor task performing experiment. Colorbar means the t-value range of the statistical parametric maps shown here. (DOCX) [file pone.0089948.s002.docx]

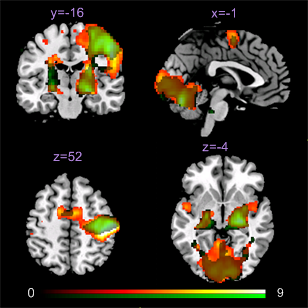


Fig. S2. Brain activations identified using standard general linear model. The task activation was defined as the significant brain activity magnitude between the task condition and control condition. The significance level used here is the same as that in Fig. 1C. Red and green indicate results of the first and second day sensorimotor task performing experiment. Colorbar means the t-value range of the statistical parametric maps shown here.
