# Supplementary material for: Brain Entropy Mapping Using fMRI
Source: PLoS One. 2014 Mar 21;9(3):e89948. doi: 10.1371/journal.pone.0089948 (PMC3962327; doi:10.1371/journal.pone.0089948)
Supplement: Figure S3 — Mean resting BEN maps of 1049 subjects calculated using SampEn with m = 3, r = 0.4, 0.6, 0.8, 1 from the bottom row to the top row, respectively. For the purpose of display, every BEN map has been normalized to be from 0 to 1 (divided by its maximum intensity). The colormap shows the display window used for generating the maps and its range is from 80% to 101% of the maximum. (DOCX) [file pone.0089948.s003.docx]

**
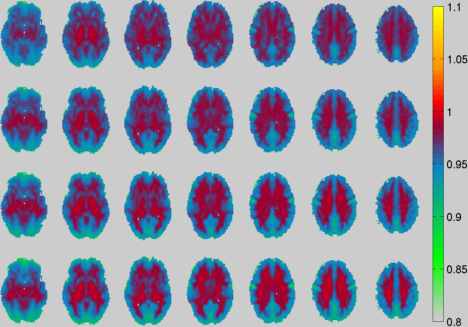
**

Fig. S3. Mean resting BEN maps of 1049 subjects calculated using SampEn with m=3, r=0.4, 0.6, 0.8, 1 from the bottom row to the top row, respectively. For the purpose of display, every BEN map has been normalized to be from 0 to 1 (divided by its maximum intensity). The colormap shows the display window used for generating the maps and its range is from 80% to 101% of the maximum.
