# Supplementary material for: Brain Entropy Mapping Using fMRI
Source: PLoS One. 2014 Mar 21;9(3):e89948. doi: 10.1371/journal.pone.0089948 (PMC3962327; doi:10.1371/journal.pone.0089948)
Supplement: Figure S4 — Silhouette coefficient (red line) and the number of non-reproducible clusters (blue line) of BEN clustering using different pre-specified cluster numbers. (DOCX) [file pone.0089948.s004.docx]

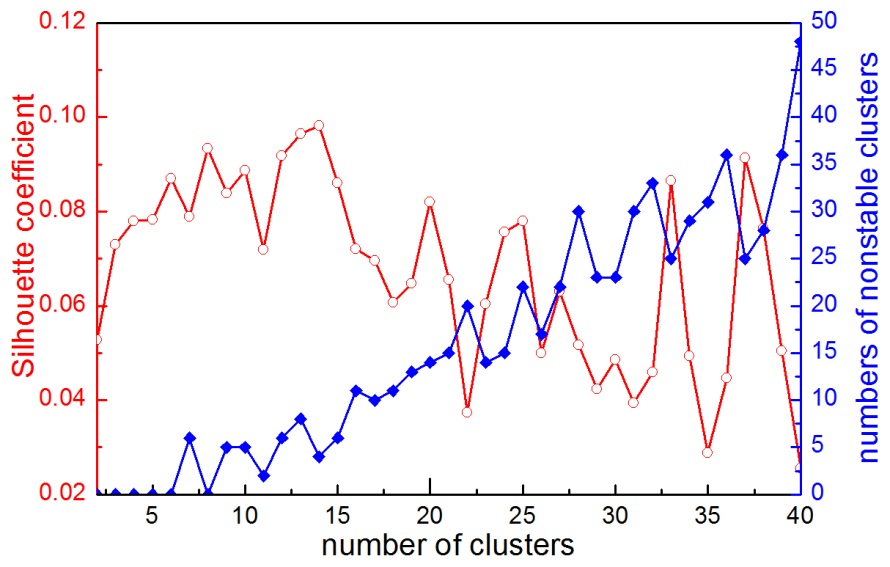


Fig. S4. Silhouette coefficient (red line) and the number of non-reproducible clusters (blue line) of BEN clustering using different pre-specified cluster numbers.
