# Supplementary material for: Brain Entropy Mapping Using fMRI
Source: PLoS One. 2014 Mar 21;9(3):e89948. doi: 10.1371/journal.pone.0089948 (PMC3962327; doi:10.1371/journal.pone.0089948)
Supplement: Figure S5 — Two different data subsets-based BEN clustering results. A) Eight brain subdivisions identified from the 525 randomly selected subjects' rBEN maps, B) eight brain subdivisions the rest 524 subjects' rBEN maps using the data-driven spectral clustering method. These clusters were identified at the optimal peak of the curves shown in Fig. S4 using the same spectral clustering procedure as described in the main article. (DOCX) [file pone.0089948.s005.docx]

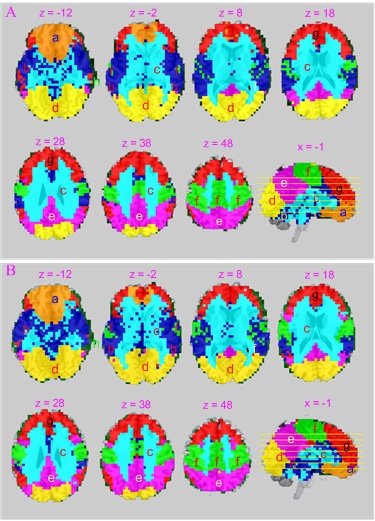


Fig. S5. Two different data subsets-based BEN clustering results. A) Eight brain subdivisions identified from the 525 randomly selected subjects’ rBEN maps, B) eight brain subdivisions the rest 524 subjects’ rBEN maps using the data-driven spectral clustering method. These clusters were identified at the optimal peak of the curves shown in Fig. S4 using the same spectral clustering procedure as described in the main article.
