# Supplementary material for: Brain Entropy Mapping Using fMRI
Source: PLoS One. 2014 Mar 21;9(3):e89948. doi: 10.1371/journal.pone.0089948 (PMC3962327; doi:10.1371/journal.pone.0089948)
Supplement: Figure S6 — A) six brain subdivisions and B) fourteen brain subdivisions derived from 1049 subjects' rBEN maps. These clusters were identified using the same spectral clustering procedure as described in the main article. The prior specified cluster number k of 6 and 14were located in the two suboptimal peak locations adjacent to the optimal one k = 8 as shown in Fig. S4. (DOCX) [file pone.0089948.s006.docx]

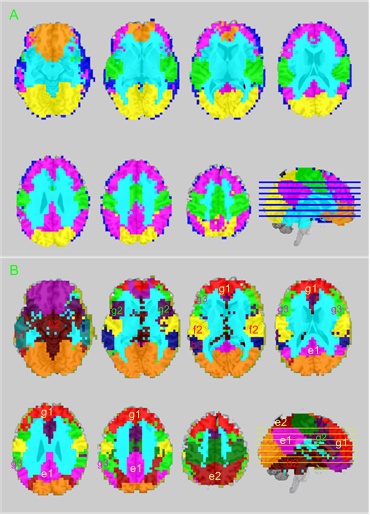


Fig. S6. A) six brain subdivisions and B) fourteen brain subdivisions derived from 1049 subjects’ rBEN maps. These clusters were identified using the same spectral clustering procedure as described in the main article. The prior specified cluster number k of 6 and 14 were located in the two suboptimal peak locations adjacent to the optimal one k=8 as shown in Fig. S4.
