# Supplementary material for: Brain Entropy Mapping Using fMRI
Source: PLoS One. 2014 Mar 21;9(3):e89948. doi: 10.1371/journal.pone.0089948 (PMC3962327; doi:10.1371/journal.pone.0089948)
Supplement: Table S1 — Signal detection results using sample entropy and reference guided data fitting. Synthetic data were generated by adding noise to a boxcar function with 8 different level of contrast-to-noise-ratio (CNR). SampEn was calculated using an embedded dimension of 4, and 5 different tolerance levels. cc means Pearson's correlation coefficient. (DOCX) [file pone.0089948.s007.docx]

Supplementary Table ST1. Signal detection results using sample entropy and reference guided data fitting. Synthetic data were generated by adding noise to a boxcar function with 8 different level of contrast-to-noise-ratio (CNR). SampEn was calculated using an embedded dimension of 4, and 5 different tolerance levels. cc means Pearson’s correlation coefficient.

|  | CNR0.08 | CNR0.1 | CNR0.2 | CNR0.5 | CNR0.8 | CNR1 |
| --- | --- | --- | --- | --- | --- | --- |
| tolerance | p-values of the SampEns difference between noise and noise contaminated signal | | | | | |
| 0.2 | 2.61E-01 | 1.30E-01 | 4.71E-04 | 6.15E-39 | 1.90E-53 | 2.26E-67 |
| 0.4 | 3.40E-03 | 2.02E-03 | 5.51E-16 | 8.03E-63 | 6.17E-78 | 1.63E-87 |
| 0.6 | 1.52E-06 | 4.75E-09 | 1.83E-27 | 9.69E-67 | 1.95E-79 | 1.98E-92 |
| 0.8 | 1.36E-07 | 7.19E-15 | 2.77E-27 | 5.05E-65 | 1.22E-77 | 1.16E-88 |
| 1 | 3.77E-07 | 8.80E-17 | 1.78E-24 | 8.49E-59 | 6.57E-75 | 1.04E-84 |
| 1.2 | 2.34E-06 | 1.71E-15 | 9.16E-25 | 2.67E-56 | 3.27E-70 | 2.61E-75 |
| 1.5 | 1.13E-04 | 2.24E-13 | 9.80E-22 | 6.40E-51 | 8.51E-56 | 5.56E-49 |
